# Kinetic data of extraction of cyanide during the soaking process of cassava leaves

**DOI:** 10.1016/j.dib.2019.104279

**Published:** 2019-07-17

**Authors:** Mohamed Hawashi, Christa Sitania, Claudya Caesy, Hakun Wirawasista Aparamarta, Tri Widjaja, Setiyo Gunawan

**Affiliations:** Department of Chemical Engineering, Institut Teknologi Sepuluh Nopember (ITS), Surabaya 60111, Indonesia

**Keywords:** Cassava leaves, Cyanide, Detoxification, Protein content, Modeling, Diffusivity

## Abstract

With the toxicity problems arising from the consumption of hydrogen cyanide (HCN), an acceptable method of processing edible leaves with low HCN level while maintaining maximum nutritional content remain a challenge. This data focuses on the extraction kinetics of cyanide in cassava leaves during the soaking process. Various process parameters conducted at 26 ± 2 °C were evaluated, such as contact times (1–20 h) between the leaves and solvent, as well as the water-to-leaves ratios (spanning a range of 10–50 mL/g). After ten h of extraction with water, all experiments resulted in less than 9.5 ppm of HCN, which is less than the toxicity level recommended by the World Health Organization (10 ppm). The mild approach resulted in protein loss from 36.01% to 23.10–25.38%. Water-to-leaves ratios of 10, 30 and 50 mL/g resulted in a calculated effective cyanide diffusion coefficient of 0.864 × 10^−13^, 1.39 × 10^−13^, and 1.61 × 10^−13^ m^2^/s. The experimental data were also analyzed using empirical mathematical models to depict the leaching process. Accordingly, the data was predominantly fitted by Diffusion approach and Verma models with coefficients of determination (R^2^) of 0.999.

**Table:** Specifications Table

Subject area	Chemical engineering
More specific subject area	Food Processing
Type of data	Tables, figures
How data was acquired	After the soaking process, the cyanide acid was determined using a titration method (SNI standard). The protein content was determined using the Kjeldahl method (AOAC international standard). Furthermore, the experimental data obtained for the cyanide content of the samples of cassava leaves was converted to the cyanide ratio for the kinetic analysis of cyanide release from cassava leaves. The mathematical models were assessed using the statistical parameters of the root mean square error (RMSE), the sum square error (SSE) and the determination coefficient (R^2^).
Data format	Raw and analyzed data
Experimental factors	All experiments were conducted at 26 ± 2 ºC room temperature. Fresh leaves were chopped and pounded in a mortar and pestle for 5 min, dried and sieved. The leaves (100 g) were soaked in a different amount of water (1000–5000 mL) over periods of time (1–20 h).
Experimental features	After each period, the cassava leaves were recovered using a sieve and filtered using a filter paper. The experimental design included experimental and kinetic investigations.
Data source location	Institute of technology Sepuluh Nopember, Surabaya, Indonesia
Data accessibility	The data is provided in this article.

**Table:** 

**Value of the Data** 1- The data provided a kinetic analysis of cyanide extraction from cassava leaves with several empirical models. 2- The acquired data will be providing useful information for the design and optimization of the water-based extraction process of cyanide from the cassava leaves. 3- The data can be used to predict the diffusion phenomena and thus, estimate the soaking time required for the removed cyanide to reach the equilibrium state. 4- This data has provided an alternative way to reduce the cyanide content by optimizing the soaking condition, without sacrificing the highly desired proteins. 5- The obtained data can be useful for future similar studies on diffusion mass transfer related to the soaking process of cassava leaves, including simultaneous effects of moderate temperature and stirring.

## 1 Data

**Fig. 1:**
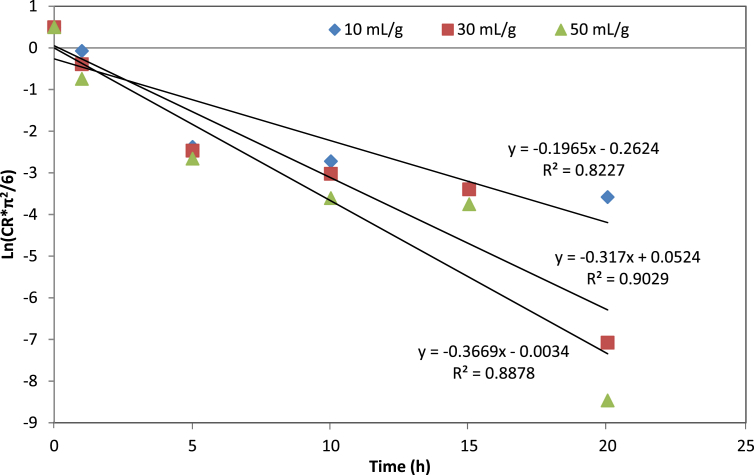
The first-order plot of cyanide extraction in cassava leaves at different water-to-leaves-ratios.

**Fig. 2:**
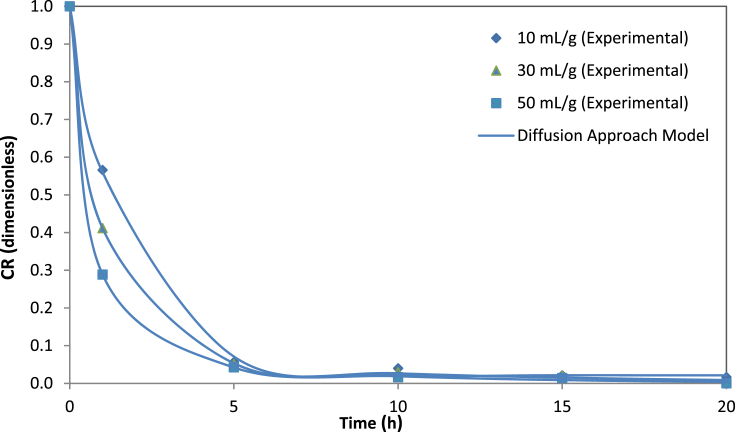
The experimental data of cyanide ratios by the Diffusion model at different water-to-leaves-ratios.

**Fig. 3:**
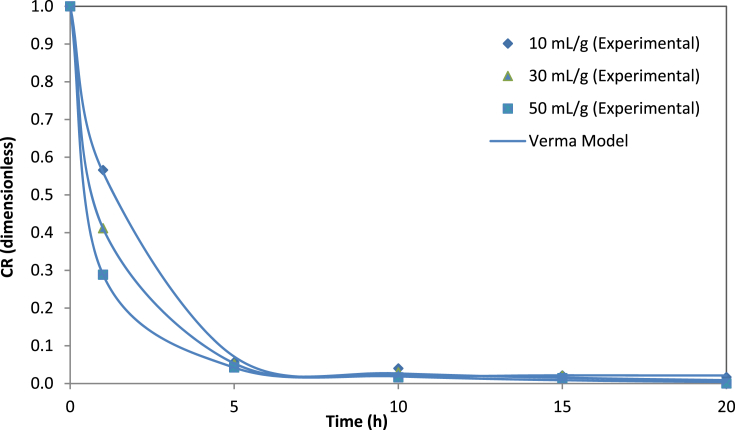
The experimental data of cyanide ratios by the Verma model at different water-to-leaves-ratios.

**Fig. 4:**
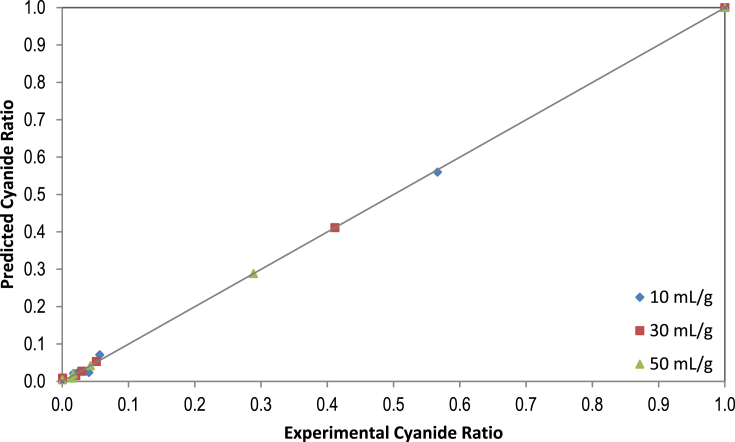
Predicted values vs. experimental values for cyanide ratios by the Diffusion model at different water-to-leaves-ratios.

**Fig. 5:**
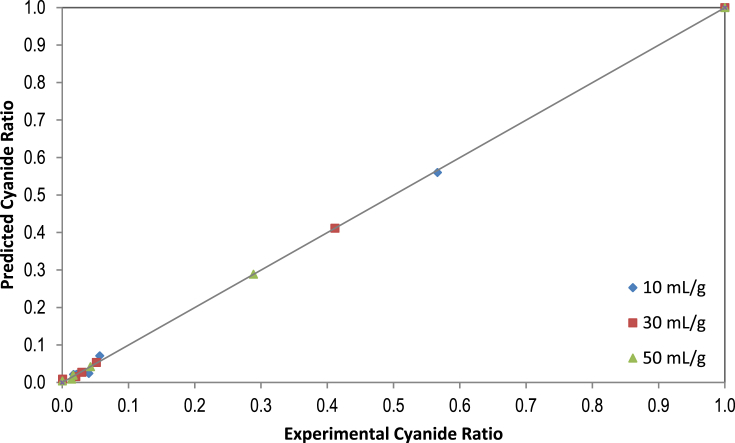
Predicted values vs. experimental values for cyanide ratios by the Verma model at different water-to-leaves-ratios.

The removal efficiency rate of cyanide content in processed leaves during the soaking process as a function of the extraction time at 10, 30, and 50 mL/g are presented in Table 1. The effect of the cassava leaf processing on the protein contents is presented in Table 2. The first-order plot of cyanide extraction in cassava leaves at 10, 30, and 50 mL/g is shown in Fig. 1. The mathematical models and their equations used are shown in Table 3. The average values and standard errors of the parameters calculated for all mathematical models used are summarized in Table 4. The values of the statistical parameters calculated for all proposed models were summarized in Table 5. The experimental cyanide ratios by the Diffusion model and Verma model at different water-to-leaves ratios are presented in Fig. 2 and Fig. 3. Comparison of predicted versus experimental cyanide ratios by the Diffusion model and Verma model at different water-to-leaves ratios are presented in Fig. 4, Fig. 5.

**Table 1:** Reduction of cyanide concentration over time at different water-to-leaves-ratios.

Soaking time (h)	water-to-leaves-ratio (10 mL/g)		water-to-leaves-ratio (30 mL/g)		water-to-leaves-ratio (50 mL/g)	
	Cyanide concentration (ppm dw)	Reduction (%)	Cyanide concentration (ppm dw)	Reduction (%)	Cyanide concentration (ppm dw)	Reduction (%)
0	159.15 ± 6.02	–	159.15 ± 6.02	–	159.15 ± 6.02	–
1	91.52 ± 6.22	42.5	67.44 ± 4.58	57.6	48.27 ± 5.62	69.7
5	12.11 ± 0.43	92.4	11.34 ± 0.28	92.9	9.94 ± 0.48	93.8
10	9.54 ± 0.83	94.0	7.57 ± 0.16	95.2	5.87 ± 0.10	96.3
15	6.63 ± 0.48	95.8	6.48 ± 0.04	95.9	5.52 ± 0.13	96.5
20	5.94 ± 0.2	96.3	3.38 ± 0.4	97.8	3.32 ± 0.53	97.9

**Table 2:** Effect of the cassava leaf processing on the protein content.

Soaking time (h)	water-to-leaves-ratio (10 mL/g)		water-to-leaves-ratio (30 mL/g)		water-to-leaves-ratio (50 mL/g)	
	DM (%)	Protein content (% dw)	DM (%)	Protein content (% dw)	DM (%)	Protein content (% dw)
0	31.76 ± 0.09	36.01 ± 1.44	31.76 ± 0.09	36.01 ± 1.44	31.76 ± 0.09	36.01 ± 1.44
1	19.58 ± 0.14	34.55 ± 0.16	19.14 ± 0.02	31.58 ± 0.93	18.86 ± 0.02	31.25 ± 0.80
5	15.23 ± 0.01	28.97 ± 0.39	15.17 ± 0.02	25.84 ± 0.34	15.13 ± 0.01	24.7 ± 0.47
10	15.14 ± 0.02	25.38 ± 1.68	15.09 ± 0.03	23.74 ± 0.61	14.97 ± 0.01	23.10 ± 0.29
15	15.08 ± 0.01	23.66 ± 0.45	14.86 ± 0.02	19.58 ± 1.22	14.66 ± 0.03	18.97 ± 0.75
20	14.79 ± 0.02	20.68 ± 0.60	14.19 ± 0.03	17.22 ± 0.54	14.03 ± 0.01	15.51 ± 0.70

**Table 3:** Empirical mathematical models used.

Model name	Model equations	References
Newton	CR=exp(−kt)	[4]
Page	$\text{CR}=\text{exp}\left(-{\text{kt}}^{\text{n}}\right)$	[5]
Logarithmic	CR=aexp(−kt)+c	[6]
Two-term	$\text{CR}=\text{a}\;\text{exp}\left(-\text{k}\begin{array}{c} \;\\ 0 \end{array}\text{t}\right)+\text{n}.\;\text{exp}\left(-\text{k}\begin{array}{c} \;\\ 1 \end{array}\text{t}\right)$	[7]
Diffusion approach	CR=aexp(−kt)+(1−a).exp(−kbt)	[8]
Verma	CR=aexp(−kt)+(1−a).exp(−bt)	[9]
Weibull	$\text{CR}=\text{exp}\left(-{\left(\frac{\text{t}}{\text{β}}\right)}^{\text{α}}\right)$	[10]

**Table 4:** Empirical parameters of proposed models used at various conditions.

Empirical model		Water-to-leaves ratio (ml/g)		
		10	30	50
Newton	k	0.5669 ± 0.0318^a^	0.8732 ± 0.0563^b^	1.2379 ± 0.0709^c^
Page	k	0.5713 ± 0.1320^a^	0.7077 ± 0.0600^a^	0.5557 ± 0.0300^b^
	n	0.9755 ± 0.0462^a^	0.8916 ± 0.0395^a^	1.2453 ± 0.0257^b^
Logarithmic	a	0.9808 ± 0.0142^a^	0.9766 ± 0.0193a	0.9820 ± 0.0189a
	k	0.5992 ± 0.0272^a^	0.9142 ± 0.0501^b^	1.2860 ± 0.0691^c^
	c	0.0216 ± 0.0071^a^	0.0225 ± 0.0088^a^	0.0178 ± 0.0085^a^
Two Term	a	0.9802 ± 0.0299^a^	0.9175 ± 0.0269^a^	0.9094 ± 0.0258^a^
	k_0_	0.5999 ± 0.0435^a^	0.9997 ± 0.0420^b^	1.4608 ± 0.0666^c^
	b	0.0226 ± 0.0275^a^	0.0824 ± 0.0261^b^	0.0906 ± 0.0253^b^
	k_1_	0.0021 ± 0.0839^a^	0.1118 ± 0.0369^b^	0.1557 ± 0.0400^c^
Diffusion approach	a	0.9781 ± 0.0227^a^	0.9175 ± 0.0213^b^	0.9094 ± 0.0206^b^
	k	−0.5973 ± 0.0324^a^	−0.9996 ± 0.0336^b^	−1.4607 ± 0.0542^c^
	b	0.0019 ± 0.1155^a^	0.1119 ± 0.0275^a^	0.1066 ± 0.019^a^
Verma	a	0.9781 ± 0.0222^a^	0.9175 ± 0.0213^b^	0.9094 ± 0.0206^b^
	k	−0.5973 ± 0.0324^a^	−0.9996 ± 0.0336^b^	−1.4607 ± 0.0542^c^
	b	−0.0011 ± 0.0690^a^	−0.1118 ± 0.0301^b^	−0.1557 ± 0.0326^c^
Weibull	α	1.7749 ± 0.134^a^	1.1759 ± 0.068^a^	0.6738 ± 0.033^b^
	β	0.9756 ± 0.103^a^	0.7078 ± 0.060^b^	0.5557 ± 0.030^c^

Different superscripts letters on the same row show different values (P < 0.05).

**Table 5:** Statistical analysis for empirical models at various conditions.

Empirical model	statistical parameters	Water-to-leaves ratio (ml/g)		
		10	30	50
Newton	RMSE	0.0203	0.0238	0.0206
	SSE	0.0020	0.0028	0.0021
	*Χ*^2^	0.00035	0.00047	0.00035
	R^2^	0.99441	0.99662	0.99738
Page	RMSE	0.0261	0.0162	0.0074
	SSE	0.0020	0.0008	0.0002
	*Χ*^2^	0.00034	0.00013	0.00001
	R^2^	0.99767	0.99902	0.99979
Logarithmic	RMSE	0.0131	0.0173	0.0170
	SSE	0.0005	0.0009	0.0008
	*Χ*^2^	0.00009	0.00015	0.00014
	R^2^	0.99939	0.99886	0.99888
Two Term	RMSE	0.0160	0.0072	0.0051
	SSE	0.0005	0.0001	0.00005
	*Χ*^2^	0.00009	0.00002	0.00001
	R^2^	0.99839	0.99987	0.99993
Diffusion approach	RMSE	0.0131	0.0058	0.0042
	SSE	0.0005	0.0001	0.00005
	*Χ*^2^	0.00009	0.00002	0.00001
	R^2^	0.99940	0.99987	0.99993
Verma	RMSE	0.0131	0.0058	0.0042
	SSE	0.0005	0.0001	0.00005
	*Χ*^2^	0.00009	0.00002	0.00001
	R^2^	0.99940	0.99987	0.99993
Weibull	RMSE	0.0261	0.0163	0.0074
	SSE	0.0020	0.0008	0.0002
	*Χ*^2^	0.00037	0.00328	0.00400
	R^2^	0.99413	0.97803	0.97227

## 2 Experimental design, materials, and methods

### 2.1 Materials

Newly harvested leaves of cassava were obtained from a cassava farm in Sidoarjo, Indonesia. All chemicals and reagents were purchased from SAP (Surabaya, Indonesia): 25% ammonia solution (NH_4_OH), 2.5% sodium hydroxide (NaOH), 5% potassium iodide (KI) solution, and 0.02N silver nitrate (AgNO_3_).

### 2.2 Sample preparation and soaking process

Fresh leaves (without petioles) were chopped and then pounded in a mortar and pestle for 5 min, thereafter dried and ground to pass through a sieve of 60 mesh size (250 μm). Immediately, 100 g of leaves were soaked in each of 1000, 3000, and 5000 mL of tap water in a bowl. Each bowl was held at 26 ± 2 °C, over periods of time (1, 5, 10, 15, and 20 h). After each period, the cassava leaves were recovered using a sieve and filtered using a filter paper. All samples were analyzed for moisture content, dry matter, cyanide, and crude protein.

### 2.3 Chemical composition analysis

The dry matter content (DM) was determined by drying the sample at 105 °C in the oven until a constant weight was reached. The content of cyanide acid in unprocessed and processed cassava leaves was determined using a titration method as described by SNI standard [1]. The protein content was determined using the Kjeldahl method (Nx6.25) as described by AOAC international standard AOAC [2]. The experimental data of unprocessed and processed cassava leaves were expressed on a dry weight basis (dw).

### 2.4 Effect of soaking conditions on cyanide concentration

To optimize the soaking process, several time periods (1, 5, 10, 15 and 20 h) and three different water-to-leaves ratios (10, 30, and 50 mL/g) were used to investigate their effect on the cyanide content, as presented in Table 1.

### 2.5 Effect of the cassava leaf processing on the protein content

The initial protein content of approximately 36.01% (dry basis) was found in cassava leaves. During the processing of cassava leaves, protein content decreased with increased contact time. Table 2 shows that the decrease in protein content in the processed leaves was less than 30% after five h of soaking in water at three the water-to-leaves ratios.

### 2.6 Determination of effective diffusivity

The experimental data obtained for the cyanide content of the samples of cassava leaves were converted to the cyanide ratio (CR) using Eq (1).

(1) $$CR=\;\frac{C_{t}-C_{e}}{C_{0}-C_{e}}$$

where C_t_, C_0_ and C_e_ are the cyanide content in real time (ppm dw), the initial and equilibrium cyanide contents (ppm dw), respectively.

The CR is used to determine the kinetics of extraction, provided that the removal of cyanide inside the solid leaves follows a diffusion mechanism. In the current experiments, the diffusion of cyanide is a dynamic process involving a change in the gradient concentration in time. As such, Fick's second law of diffusion was applied for long contact times and considered a one-dimensional diffusion of spherical molecules through the dilute solution, the first term of the series development in (Eq. (2)) [3].

(2) $$CR=\;\frac{6}{\pi^{2}}e^{-\frac{D_{eff}\;\pi^{2}}{r^{2}}t}$$

where D_eff_, t, and r are the effective cyanide diffusion coefficient (m^2^/s), the soaking time (h), and the equivalent radius (m), respectively.

The effective cyanide diffusion coefficients (D_eff_) were calculated using the slope of ln (CR) vs. time, as illustrated in Fig. 1. The effective cyanide diffusion coefficient values at different water-to-leaves ratios (i.e., 10, 30, and 50 mL/g) calculated by linear regression method were (8.64 ± 0.51) x 10^−14^, (1.39 ± 0.06) x 10^−13^, and (1.61 ± 0.07) x 10^−13^ m^2^/s, respectively.

### 2.7 Mathematical models of cyanide extraction kinetics

The cyanide ratio data obtained from three different water-to-leaves ratios were fitted into seven mathematical models which are shown in Table 3. The models proposed here to evaluate the cyanide extraction kinetics mainly considered the difference of residual cyanide content as a function of time. The parameters calculated for proposed mathematical models are shown in Table 4.Where CR is cyanide ratio, t is the soaking time (h), and a, n, α, c, and β are the empirical parameters.

The suitability of the mathematical models was assessed using the statistical parameters of the root mean square error (RMSE), the sum square error (SSE), the chi-square (Χ^2^) and the determination coefficient (R^2^) using Eqs. (3), (4), (5), (6) [11], [12]:

(3) $$RMSE=\sqrt{\left[{\frac{1}{N}\overset{N}{\underset{j=1}{\sum }}\left(C_{ej}-C_{pj}\right)}^{2}\right]}$$

(4) $${SSE=\frac{1}{N}\overset{N}{\underset{j=1}{\sum }}\left(C_{ej}-C_{pj}\right)}^{2}$$

(5) $$\chi^{2}=\frac{\sum_{i=1}^{N}{\left(C_{ej}-C_{pj}\right)}^{2}}{N-n}$$

(6) $$R^{2}=1-\frac{{\sum_{i=1}^{N}\left(C_{ej}-C_{pj}\right)}^{2}}{{{\sum_{i=1}^{N}\left(C_{pj}-\bar{C_{ej}}\right)}^{2}+\sum_{i=1}^{N}\left(C_{ej}-C_{pj}\right)}^{2}}$$

where C_ej_ is the experimental cyanide ratio (−), C_pj_ is the predicted cyanide ratio (−), N is the number of observations, and j is the number of terms.

The kinetic data have been analyzed by regression analysis using Minitab 18.1 statistical software.

Statistical parameters such as RMSE, SSE, X ^2^and R^2^ are common practice in the kinetic studies, which provide useful information about the suitability of mathematical models for the experimental data [9]. The values of the statistical parameters calculated for all proposed models are presented in Table 5. The phenomenon of cyanide mass transfer (by diffusion) under different experimental conditions obeys empirical or diffusion models with values close to zero for RMSE, SSE and X^2^. In addition, the regression coefficients of R^2^ were above 0.97 for all models, which suggests that all the proposed models were suitable for experimental data. The lowest SSE, RMSE and X^2^ values and the highest R^2^ values were used to choose the best model. If these statistical tests applied are considered at three different water-to-leaves ratios (i.e., 10, 30, and 50 mL/g), the model equation that best fitted the experimental cyanide leaching data proved to be the Diffusion Approach and Verma models, as shown in Fig. 2, Fig. 3. For validation of the selected models (Diffusion Approach and Verma), experimental data for cyanide ratios were compared with the predicted data at three different water-to-leaves ratios, as shown in Fig. 4, Fig. 5. The diagnostic plots showed that the experimental data was very similar to the predicted data by these models. Thus, these two models adequately describe the experimental data of cyanide leaching at three different water-to-leaves ratios.
