# Supplementary material for: Kinetic data of extraction of cyanide during the soaking process of cassava leaves
Source: Data Brief. 2019 Jul 17;25:104279. doi: 10.1016/j.dib.2019.104279 (PMC6685679; doi:10.1016/j.dib.2019.104279)
Supplement: Multimedia component 1 [file mmc1.docx]

| Time  (h) | Moisture content (%) | | |
| --- | --- | --- | --- |
|  | 10 mL/g | 10 mL/g | Average |
| **0** | 67.61 | 66.46 | 67.03 |
| 1 | 76.09 | 78.10 | 77.09 |
| 5 | 79.10 | 85.27 | 82.18 |
| 10 | 80.20 | 83.45 | 81.82 |
| 15 | 79.75 | 86.63 | 83.19 |
| 20 | 79.28 | 85.30 | 82.29 |

| Time  (h) | Moisture content (%) | | |
| --- | --- | --- | --- |
|  | 30 mL/g | 30 mL/g | Average |
| **0** | 67.61 | 66.46 | 67.03 |
| 1 | 78.24 | 79.39 | 78.81 |
| 5 | 81.97 | 85.71 | 83.84 |
| 10 | 76.67 | 87.14 | 81.90 |
| 15 | 78.37 | 85.24 | 81.80 |
| 20 | 79.27 | 89.15 | 84.21 |

| Time  (h) | Moisture content (%) | | |
| --- | --- | --- | --- |
|  | 50 mL/g | 50 mL/g | Average |
| **0** | 67.61 | 66.46 | 67.03 |
| 1 | 77.21 | 81.67 | 79.44 |
| 5 | 80.55 | 85.57 | 83.06 |
| 10 | 76.81 | 86.60 | 81.70 |
| 15 | 75.51 | 84.23 | 79.87 |
| 20 | 80.51 | 85.72 | 83.11 |

| Time  (h) | HCN content (% ww) | |
| --- | --- | --- |
|  | 10 mL/g | 10 mL/g |
| **0** | 54.456 | 50.487 |
| 1 | 22.392 | 19.542 |
| 5 | 2.234 | 2.081 |
| 10 | 1.583 | 1.885 |
| 15 | 1.033 | 1.195 |
| 20 | 1.016 | 1.087 |

| Time  (h) | HCN content (% ww) | |
| --- | --- | --- |
|  | 30 mL/g | 30 mL/g |
| **0** | 54.456 | 50.487 |
| 1 | 13.320 | 15.261 |
| 5 | 1.877 | 1.787 |
| 10 | 1.341 | 1.399 |
| 15 | 1.186 | 1.172 |
| 20 | 0.470 | 0.596 |

| Time  (h) | HCN content (% ww) | | |
| --- | --- | --- | --- |
|  | 50 mL/g | 50 mL/g |  |
| **0** | 54.456 | 50.487 |  |
| 1 | 11.079 | 8.768 |  |
| 5 | 1.602 | 1.765 |  |
| 10 | 1.092 | 1.055 |  |
| 15 | 1.139 | 1.082 |  |
| 20 | 0.471 | 0.650 |  |

| Time  (h) | HCN content (% dw) | | |
| --- | --- | --- | --- |
|  | 10 mL/g | 10 mL/g | Average |
| 0 | 165.17 | 153.13 | 159.15 |
| 1 | 97.74 | 85,3 | 91.52 |
| 5 | 12.54 | 11.68 | 12.11 |
| 10 | 8.71 | 10.37 | 9.54 |
| 15 | 6.15 | 7.11 | 6.63 |
| 20 | 5.74 | 6.14 | 5.94 |

| Time  (h) | HCN content (% dw) | | |
| --- | --- | --- | --- |
|  | 30 mL/g | 30 mL/g | Average |
| **0** | 165.17 | 153.13 | 159.15 |
| 1 | 62.86 | 72.02 | 67.44 |
| 5 | 11.62 | 11.06 | 11.34 |
| 10 | 7.41 | 7.73 | 7.57 |
| 15 | 6.52 | 6.44 | 6.48 |
| 20 | 2.98 | 3.78 | 3.38 |

| Time  (h) | HCN content (% dw) | | |
| --- | --- | --- | --- |
|  | 50 mL/g | 50 mL/g | Average |
| **0** | 165.17 | 153.13 | 159.15 |
| 1 | 53.89 | 42.65 | 48.27 |
| 5 | 9.46 | 10.42 | 9.94 |
| 10 | 5.97 | 5.77 | 5.87 |
| 15 | 5.66 | 5.38 | 5.52 |
| 20 | 2.79 | 3.85 | 3.32 |

| Time  (h) | Protein content (% ww) | | |
| --- | --- | --- | --- |
|  | 10 mL/g | 10 mL/g |  |
| **0** | 11.397 | 12.347 |  |
| 1 | 8.055 | 7.775 |  |
| 5 | 5.231 | 5.092 |  |
| 10 | 4.919 | 4.308 |  |
| 15 | 4.052 | 3.901 |  |
| 20 | 3.768 | 3.556 |  |

| Time  (h) | Protein content (% ww) | | |
| --- | --- | --- | --- |
|  | 30 mL/g | 30 mL/g |  |
| **0** | 11.397 | 12.347 |  |
| 1 | 6.494 | 6.888 |  |
| 5 | 4.230 | 4.120 |  |
| 10 | 4.186 | 4.407 |  |
| 15 | 3.785 | 3.341 |  |
| 20 | 2.633 | 2.804 |  |

| Time  (h) | Protein content (% ww) | | |
| --- | --- | --- | --- |
|  | 50 mL/g | 50 mL/g |  |
| **0** | 11.397 | 12.347 |  |
| 1 | 6.260 | 6.589 |  |
| 5 | 4.104 | 4.263 |  |
| 10 | 4.280 | 4.174 |  |
| 15 | 3.667 | 3.969 |  |
| 20 | 2.737 | 2.501 |  |

| Time  (h) | Protein content (% dw) | | |
| --- | --- | --- | --- |
|  | 10 mL/g | 10 mL/g | Average |
| **0** | 34.57 | 37.45 | 36.01 |
| 1 | 35.16 | 33.94 | 34.55 |
| 5 | 29.36 | 28.58 | 28.97 |
| 10 | 27.06 | 23.7 | 25.38 |
| 15 | 24.11 | 23.21 | 23.66 |
| 20 | 21.28 | 20.08 | 20.68 |

| Time  (h) | Protein content (% dw) | | |
| --- | --- | --- | --- |
|  | 30 mL/g | 30 mL/g | Average |
| **0** | 34.57 | 37.45 | 36.01 |
| 1 | 30.65 | 32.51 | 31.58 |
| 5 | 26.18 | 25.5 | 25.84 |
| 10 | 23.13 | 24.35 | 23.74 |
| 15 | 20.8 | 18.36 | 19.58 |
| 20 | 16.68 | 17.76 | 17.22 |

| Time  (h) | Protein content (% dw) | | |
| --- | --- | --- | --- |
|  | 50 mL/g | 50 mL/g | Average |
| **0** | 34.57 | 37.45 | 36.01 |
| 1 | 30.45 | 32.05 | 31.25 |
| 5 | 24.23 | 25.17 | 24.7 |
| 10 | 23.39 | 22.81 | 23.10 |
| 15 | 18.22 | 19.72 | 18.97 |
| 20 | 16.21 | 14.81 | 15.51 |

| Time  (h) | DM % | | |
| --- | --- | --- | --- |
|  | 10 mL/g | 10 mL/g | Average |
| **0** | 31.85 | 31.67 | 31.76 |
| 1 | 19.73 | 19.44 | 19.58 |
| 5 | 15.22 | 15.24 | 15.23 |
| 10 | 15.16 | 15.12 | 15.14 |
| 15 | 15.08 | 15.09 | 15.08 |
| 20 | 14.81 | 14.77 | 14.79 |

| Time  (h) | DM % | | |
| --- | --- | --- | --- |
|  | 30 mL/g | 30 mL/g | Average |
| **0** | 31.85 | 31.67 | 31.76 |
| 1 | 19.13 | 19.16 | 19.14 |
| 5 | 15.15 | 15.19 | 15.17 |
| 10 | 15.06 | 15.12 | 15.09 |
| 15 | 14.88 | 14.84 | 14.86 |
| 20 | 14.22 | 14.16 | 14.19 |

| Time  (h) | DM % | | |
| --- | --- | --- | --- |
|  | 50 mL/g | 50 mL/g | Average |
| **0** | 31.85 | 31.67 | 31.76 |
| 1 | 18.88 | 18.84 | 18.86 |
| 5 | 15.12 | 15.14 | 15.13 |
| 10 | 14.96 | 14.98 | 14.97 |
| 15 | 14.63 | 14.69 | 14.66 |
| 20 | 14.02 | 14.04 | 14.03 |
